# High mortality rates among COVID-19 intensive care patients in Iraq: insights from a retrospective cohort study at Médecins Sans Frontières supported hospital in Baghdad

**DOI:** 10.3389/fpubh.2023.1185330

**Published:** 2023-08-31

**Authors:** Rami Malaeb, Amna Haider, Mustafa Abdulateef, Mustafa Hameed, Uche Daniel, Gabriel Kabilwa, Ibrahim Seyni, Khalid E. Ahmadana, Evgenia Zelikova, Klaudia Porten, Aurelie Godard

**Affiliations:** ^1^Department of Epidemiology and Training, Epicentre, Dubai, United Arab Emirates; ^2^Rusafa Directorate of Health, Baghdad, Iraq; ^3^Médecins Sans Frontières, Operational Centre Paris, Baghdad, Iraq; ^4^Médecins Sans Frontières, Operational Centre Paris, Dubai, United Arab Emirates; ^5^Médecins Sans Frontières, Operational Centre Paris, Paris, France; ^6^Department of Epidemiology and Training, Epicentre, Paris, France

**Keywords:** ICU outcomes, COVID-19, Iraq, Baghdad, ICU mortality, healthcare, limited resource settings, humanitarian

## Abstract

**Background:**

The Coronavirus Disease 2019 (COVID-19) pandemic has highlighted the challenges of the healthcare system in Iraq, which has limited intensive care unit beds, medical personnel, and equipment, contributing to high infection rates and mortality. The main purpose of the study was to describe the clinical characteristics, the length of Intensive Care Unit (ICU) stay, and the mortality outcomes of COVID-19 patients admitted to the ICU during the first wave and two subsequent surges, spanning from September 2020 to October 2021, in addition to identify potential risk factors for ICU mortality.

**Methods:**

This retrospective cohort study analyzed data from COVID-19 patients admitted to the COVID-19 ICU at Al-Kindi Ministry of Health hospital in Baghdad, Iraq, between September 2020 and October 2021.

**Results:**

The study included 936 COVID-19 patients admitted to the ICU at Al-Kindi Hospital. Results showed a high mortality rate throughout all waves, with 60% of deaths due to respiratory failure. Older age, male gender, pre-existing medical conditions, ICU procedures, and complications were associated with increased odds of ICU mortality. The study also found a decrease in the number of complications and ICU procedures between the first and subsequent waves. There was no significant difference in the length of hospital stay between patients admitted during different waves.

**Conclusion:**

Despite improvements in critical care practices, the mortality rate did not significantly decrease during the second and third waves of the pandemic. The study highlights the challenges of high mortality rates among critical COVID-19 patients in low-resource settings and the importance of effective data collection to monitor clinical presentations and identify opportunities for improvement in ICU care.

## Background

Since the start of the outbreak of the coronavirus disease 2019 (COVID-19), critical patients have required advanced level of intensive medical care (1). The global pandemic has placed a significant strain on the availability of intensive care unit (ICU) beds, leading many countries to implement strategies to improve access and efficiency of intensive care. COVID-19 patients admitted to the ICU need a prolonged hospital stay under a treatment regimen that includes anti-viral or steroid therapy, together with supplemental oxygen often through invasive mechanical ventilation (IMV) (2–4). During the course of this pandemic, low- and middle-income countries (LMICs) were particularly disadvantaged due to their limited resources and difficulty in rapidly expanding ICU bed capacity, providing sufficient oxygen, and maintaining quality of care (5).

The in-hospital mortality rates among critically ill COVID-19 patients remained high throughout the course of the pandemic. A systematic review and meta-analysis of 52 studies revealed an overall ICU mortality rate of 35.5% for COVID-19 patients. The Middle East region had the highest reported mortality rate at 61.9% (1). Older age, male gender, smoking, obesity, co-existing conditions, and complications have been established as major risk factors for increased severity and mortality of COVID-19 globally (6–10). The magnitude of these risk factors is influenced by the context and underlying patient characteristics and clinical conditions.

Iraq was especially vulnerable during the COVID-19 pandemic due to its inadequate healthcare system and limited capacity (11, 12). At the time of the COVID-19 declaration, the country had less than 1,000 ICU beds and continued to struggle with shortages in medical personnel and equipment (13, 14). The pandemic also resulted in a shortage in oxygen supply forcing patients to transfer management to their homes instead of hospital. Additionally, the infection prevention and control measures in Iraq were inadequate, leading to high numbers of cases among healthcare workers and deterring patients from seeking care (15). Over the course of 2020 to 2021, over two million confirmed COVID-19 cases and 24,000 deaths were reported in Iraq (16). Studies from Iraq reported high infection rates among males and predominantly among those aged 30–60 years (14). Furthermore, reports have shown that individuals with chronic conditions, older age, and male gender have a higher risk of mortality, which aligns with the established risk factors globally (17–19).

The Al Kindi Hospital in Baghdad, a tertiary facility under the jurisdiction of the Ministry of Health (MOH), was designated as a center for the isolation and treatment of patients with suspected or confirmed cases of SARS-CoV-2. From July 2020 to October 2021, the Intensive Care Unit was operated with support from Médecins Sans Frontières (MSF), also known as Doctors Without Borders, offering the most optimal medical care and limited to non-invasive mechanical ventilation (Continuous Positive Airway Pressure (CPAP), Flow O₂ 25, and ICU ventilators). Invasive mechanical ventilation was not provided, in accordance with the MOH mandate. The focus of the MSF collaboration was to support and improve ICU management, starting with a capacity of 24 beds in September 2020 and gradually increasing to 55 beds by the end of October 2021.

The present study describes the clinical characteristics, the length of Intensive Care Unit (ICU) stay, and the mortality outcomes of COVID-19 patients admitted to the ICU during the first wave and two subsequent surges, spanning from September 2020 to October 2021. The aim of this analysis is to identify potential risk factors for ICU mortality among patients, and to evaluate if there was any improvement in the quality of care, as evidenced by decreased complications, shorter ICU duration, and reduced mortality rate across various stages of the epidemic in Iraq.

## Methods

### Study design

This study is a retrospective cohort study that analyzed data from confirmed and suspected COVID-19 patients admitted to the COVID-19 ICU at Al-Kindi MOH Hospital in Baghdad, Iraq between September 26, 2020 and October 13, 2021.

### Study setting and population

**Figure 1:**
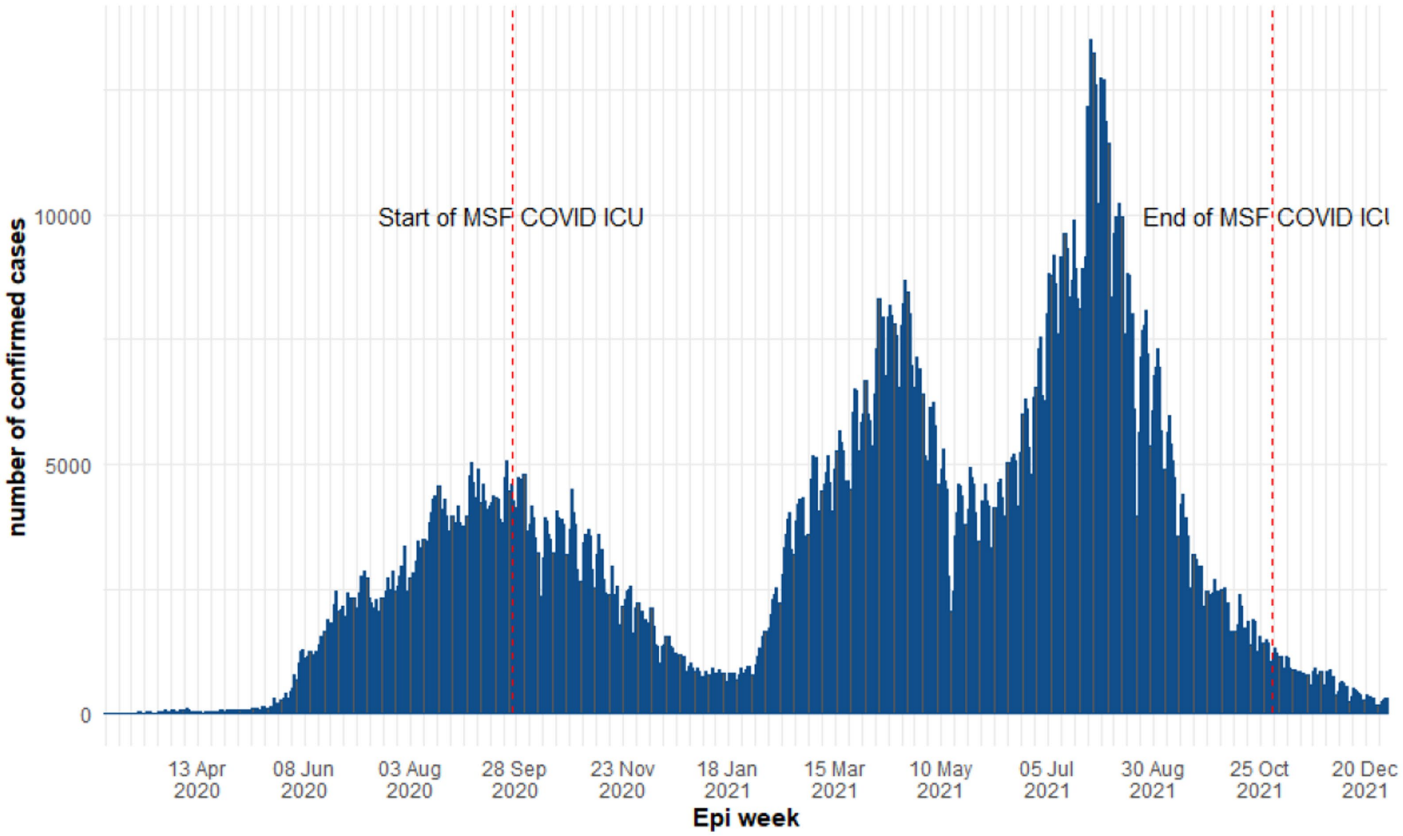
Epidemiological curve of COVID-19 daily cases in Iraq between 2020 and 2021 (World Health Organization).

The first confirmed case reported in Iraq was on 24 February 2020 in the Najaf governorate [15]. Consequently, Iraq experienced the first COVID-19 wave between February 2020 and January 2021 reaching a weekly peak of 30,059 confirmed cases and 708 deaths in week 37. The two subsequent waves were recorded in 2021; wave two from 18 January to 17 May 2021 and wave three from 24 May too 27 December 2021 recordeded weekly peaks of 54,301 and 83,098 cases including 281 and 522 deaths, respectively (Figure 1) (20). Accordingly, the study period was divided into three time periods based on the three waves of COVID-19 in Iraq and during which patients were admitted to the MSF supported COVID-19 ICU in Al-Kindi MOH Hospital. The follow-up time was defined from ICU admission to discharge, referral or death.

In this study, we included all patients admitted during the study period and who met the clinical and epidemiological criteria (21). The clinical presentation defined as critical or severe case requiring oxygen support of 7 L/min or more to achieve oxygen saturation levels above 92%. We excluded patients who refused or failed a trial of CPAP in the ER, had a Glasgow Coma Scale (GCS) score lower than 9, were too unstable for transfer, had an untreated medical or surgical problem requiring urgent intervention, or had metastatic cancer. Only patients with complete data on survival outcomes were included in the study.

### Variables and data sources

Demographic and clinical data were collected and recorded in an Excel-based database by the ICU team as part of routine data collection. However, laboratory findings and treatment regimens provided were not recorded in the database. The severity of the disease was categorized according to the WHO definition. COVID-19 diagnosis was confirmed with a positive reverse transcription-polymerase chain reaction (RT-PCR) test or chest CT scan as determined by the clinical team.

The variables included in the database consisted of the following:

- Demographic characteristics: age and sex of the ICU patients.
- Pre-existing medical conditions: Information regarding any underlying health conditions or comorbidities reported by the study participants for the majority of conditions. As for obesity condition, it was based on BMI results, yet there was no differentiation between obesity and morbid obesity in data collection.
- ICU admission and exit dates: The dates of admission to and discharge from the intensive care unit were recorded to determine the length of ICU stay.
- Chest computerized tomogram (CT) scan findings: Findings from chest CT scans were documented to assess the characteristic features of COVID-19 infection. The primary radiological findings indicative of COVID-19 were bilateral patchy areas of ground glass infiltration/appearance, predominantly observed in lower lobes and periphery of the lungs.
- Non-invasive ventilation: Information on the use of non-invasive ventilation techniques among the COVID-19 patients.
- ICU-related procedures: Any procedures performed on the patients during their stay in the ICU.
- Complications: The occurrence of any complications or adverse events during the ICU stay.
- Outcomes: The final outcomes of the COVID-19 patients.

To ensure data quality control, the ICU team diligently recorded the data during routine patient care under the support of data manager and epidemiologist who implemented data validation techniques after entry.

### Study outcomes

The primary outcome of the study was to assess the risk factors associated with ICU mortality and length of stay. The secondary outcome was to evaluate the changes in the clinical and demographic characteristics and outcomes between the different COVID-19 waves during the study period.

### Statistical analysis

The sample size was equal to the total number of patients admitted to the ICU during the study period. Descriptive statistics were performed based on the STROBE checklist for observational studies (22). Categorical variables were presented as frequencies and percentages, and continuous variables were reported as medians (interquartile ranges). Differences between groups were assessed using chi-squared or Fisher’s exact tests for categorical variables and Student’s t-test or the Kruskal-Wallis rank sum test for continuous variables, as appropriate. Univariate and multiple logistic regression models were used to identify associations between patient characteristics and ICU mortality. A stepwise selection approach based on the Akaike Information Criterion (AIC) to identify the best-fitting regression model was used. The results of the multiple regression analysis were reported as odds ratios (OR) with their 95% confidence intervals (CI). Kaplan–Meier survival curves were plotted and were compared using log-rank test. A level of significance of *p* < 0.05 was considered statistically significant. Missing data were reported in results and were not imputed. Data analysis was performed using R Studio software, version 3.6.3.

### Ethical considerations

This study was approved by the Research Committee of the National Centre for Training and Human at the Ministry of Health and Environment, Baghdad, Iraq on 31 May 2022 (protocol number 10/2022) and was exempt from the MSF Ethics Review Board following the approval of the Medical Director.

## Results

### Baseline characteristics of the study population

**Figure 2:**
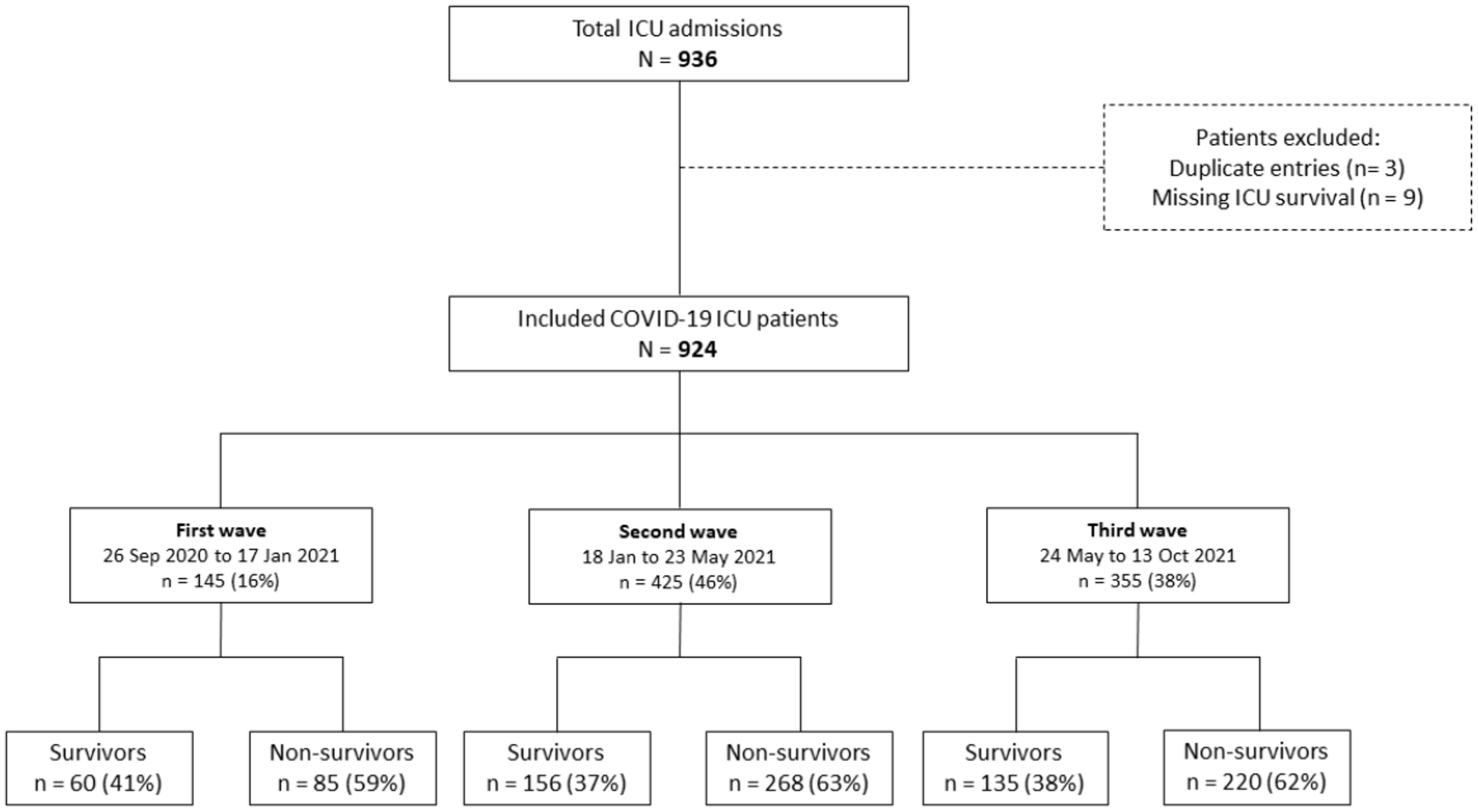
Study flowchart of COVID-19 ICU patients admitted at Al-Kindi MOH Hospital, Baghdad.

The study population consisted of 936 patients admitted to the ICU at Al-Kindi Hospital in Iraq between September 26, 2020 and October 13, 2021. After removing duplicated records and missing data, 924 patients were included in the analysis, with 145 (16%) admitted during the first wave, 425 (46%) during the second wave, and 355 (38%) during the third wave (Figure 2). The majority of patients were men (59%, *n* = 545) with a median age of 60 years (IQR 50–68) and over half of the study cohort had at least one pre-existing medical condition (55%, *n* = 511). The most common pre-existing conditions were cardiovascular disease (38%, n = 354), followed by diabetes (28%, *n* = 260) and hypertension (27%, *n* = 246) (Table 1).

**Table 1:** Baseline characteristics and clinical outcomes by COVID-19 waves of ICU patients admitted to Al-kindi MOH Hospital, Baghdad, Iraq. between 26 Sep 2020 and 13 Oct 2021

	Overall *N* = 924	1st wave *n* = 145	2nd wave *n* = 424	3rd wave *n* = 355	*p*-value^a^
**Demographics characteristics**					
Age in years, (median, IQR)	60 (50, 68)	64 (54, 71)	61 (52, 70)	56 (47, 64)	<0.001
*Missing data*	1	–	1	–	
Sex, *n* (%)					0.6
Female	379 (41%)	56 (39%)	181 (43%)	142 (40%)	
Male	545 (59%)	89 (61%)	243 (57%)	213 (60%)	
**Pre-existing medical condition** ^**b**^ **, *n* (%)**					
At least one pre-existing condition	511 (55%)	33 (23%)	263 (62%)	215 (61%)	<0.001
Cardiovascular disease	354 (38%)	18 (12%)	192 (45%)	144 (41%)	<0.001
Diabetes	260 (28%)	9 (6.2%)	135 (32%)	116 (33%)	<0.001
Hypertension	246 (27%)	11 (7.6%)	163 (38%)	72 (20%)	<0.001
Obesity	100 (11%)	5 (3.4%)	60 (14%)	35 (9.9%)	0.001
Renal disease	33 (3.6%)	6 (4.1%)	21 (5.0%)	6 (1.7%)	0.047
Lung disease	32 (3.5%)	2 (1.4%)	20 (4.7%)	10 (2.8%)	0.12
Cerebrovascular disease	27 (2.9%)	5 (3.4%)	15 (3.5%)	7 (2.0%)	0.4
Other^b^	38 (4.1%)	5 (3.4%)	20 (4.7%)	13 (3.7%)	0.7
**No. of pre-existing medical conditions, *n* (%)**					< 0.001
None	413 (45%)	112 (77%)	161 (38%)	140 (39%)	
One	179 (19%)	17 (12%)	77 (18%)	85 (24%)	
Two or more	332 (36%)	16 (11%)	186 (44%)	130 (37%)	
**COVID-19 Diagnosis, *n* (%)**					
PCR result					<0.001
Negative PCR Result	161 (21%)	35 (29%)	86 (26%)	40 (13%)	
Positive PCR Result	603 (79%)	87 (71%)	242 (74%)	274 (87%)	
*Missing data*	*160*	*23*	*96*	*41*	
CT scan result					0.086
Non-suggestive of Covid-19	367 (42%)	62 (44%)	158 (38%)	147 (46%)	
Suggestive of Covid-19	507 (58%)	79 (56%)	256 (62%)	172 (54%)	
*Missing data*	*50*	*4*	*10*	*36*	
**ICU procedures, *n* (%)**					
At least one ICU procedure	832 (90%)	124 (86%)	361 (85%)	347 (98%)	<0.001
Prone positioning	824 (89%)	119 (82%)	358 (84%)	347 (98%)	<0.001
Thoracostomy tube	18 (1.9%)	8 (5.5%)	9 (2.1%)	1 (0.3%)	<0.001
Blood transfusion	14 (1.5%)	12 (8.3%)	2 (0.5%)	0 (0%)	<0.001
Central line	14 (1.5%)	9 (6.2%)	3 (0.7%)	2 (0.6%)	<0.001
Enteral nutrition	12 (1.3%)	5 (3.4%)	7 (1.7%)	0 (0%)	0.002
Parenteral nutrition	5 (0.5%)	0 (0%)	3 (0.7%)	2 (0.6%)	0.9
Other procedures^c^	17 (1.8%)	3 (2.1%)	13 (3.1%)	1 (0.3%)	0.007
**ICU complications, *n* (%)**					
Complications	170 (18%)	52 (36%)	102 (24%)	16 (4.5%)	<0.001
Nasal sore	134 (15%)	30 (21%)	88 (21%)	16 (4.5%)	<0.001
Bed sore	55 (6%)	10 (6.9%)	37 (8.7%)	8 (2.3%)	<0.001
Urinal catheter infection	22 (2.4%)	18 (12%)	4 (0.9%)	0 (0%)	<0.001
Ventilator-associated pneumonia (clinical)	16 (1.7%)	7 (4.8%)	9 (2.1%)	0 (0%)	<0.001
Equipment failure	4 (0.4%)	3 (2.1%)	1 (0.2%)	0 (0%)	0.013
Central line infection	2 (0.2%)	2 (1.4%)	0 (0%)	0 (0%)	0.024
**Death at ICU, *n* (%)**	573(62%)	85 (61%)	268 (63%)	220 (62%)	0.6

a Kruskal-Wallis rank sum test; Pearson’s Chi-squared test; Fisher’s exact test.

b Each patient could have more than one pre-existing condition, so the sum of percentages may not add up to 100.

c Other procedures include hemodialysis (*n* = 4) and NG tube insertion (*n* = 1), the remaining other procedures were not captured comprehensively in the study database.

### Changes of characteristics and clinical outcomes between waves

Patients admitted in the second and third waves were younger (61[IQR 52,70] and 56[IQR 47,64] vs. 64 [IQR 54,71], *p* < 0.001) and had higher incidence of pre-existing medical conditions compared to the first wave, such as cardiovascular diseases (45, 41% vs. 12%, *p* < 0.001), diabetes (32, 33% vs. 6.2%, *p* < 0.001), hypertension (38, 20% vs. 7.6%, p < 0.001), and obesity (14, 10% vs. 3.4%, *p* = 0.001). The distribution of pre-existing conditions shifter across waves. In the first wave, most patients (77%) had no pre-existing conditions, whereas in the second (44%) and third waves (37%), the majority had two or more (*p* < 0.001). The RT-PCR showed a lower rate of negative results in the third wave (13% vs. 29, 26%, *p* < 0.001), while the CT scan results showed no significant difference between waves (44, 38 and 46% respectively, *p* = 0.086). Awake prone positioning was performed in over 80% of patients throughout the waves, reaching 98% in the third wave (*p* < 0.001). However, procedures such as thoracostomy, blood transfusion, and central line were significantly reduced between the first and subsequent waves (*p* < 0.001). The number of complications observed in the hospital decreased from 36% in the first to 24% in the second and 4.5% in the third wave (*p* < 0.001).

### Mortality outcomes

**Figure 3:**
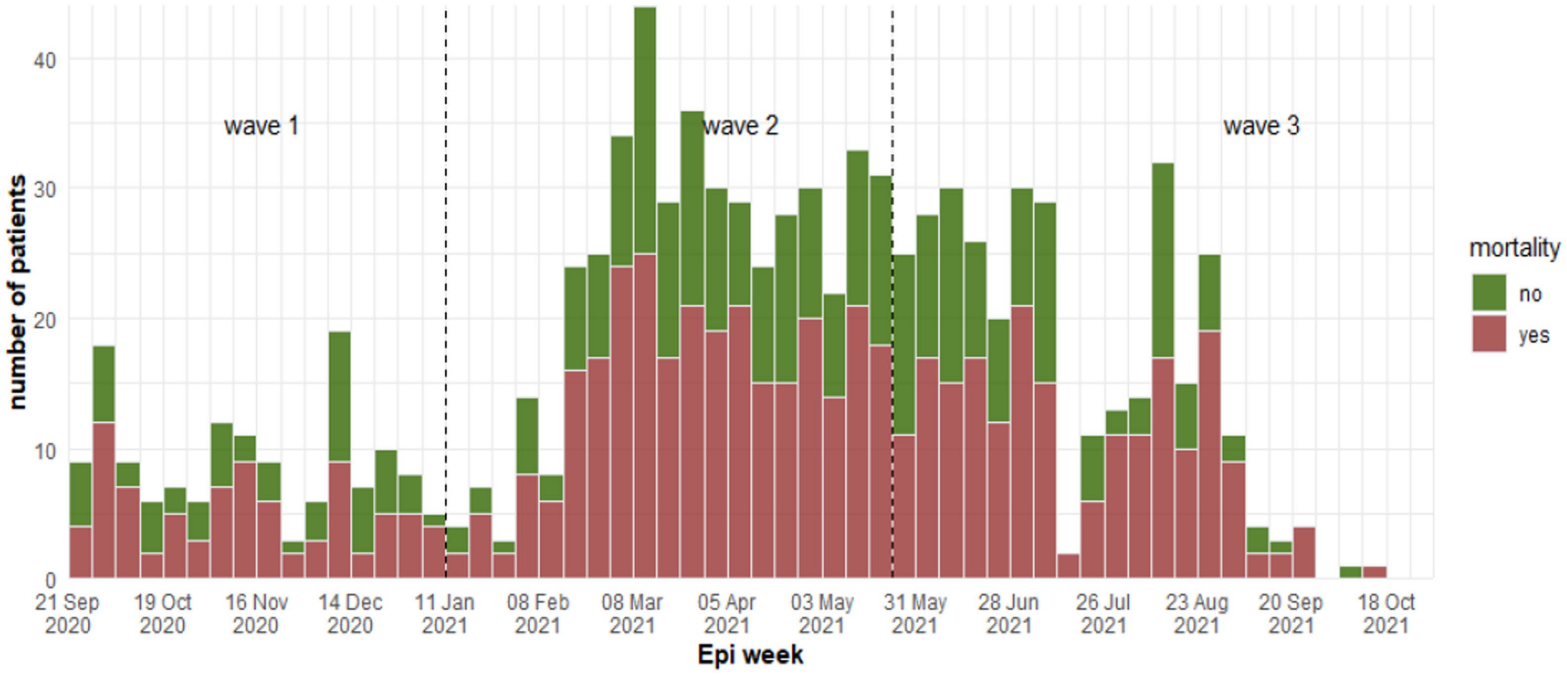
Weekly number of fatality cases between waves at Al-Kindi Hospital, Baghdad Iraq 2020–2021.

Patients were categorized as either survivors (351/924) and non-survivors (573/924) at discharge or death. The weekly death rate was consistently high throughout the three waves (Figure 3) with 60% (*n* = 345) of deaths due to respiratory failure, followed by multi-organ failure (29%, *n* = 168), septic shock (4%, *n* = 23) and cardiogenic shock (2.4%, *n* = 14). Univariate analysis is presented in Table 2.

**Table 2:** Comparison between survivors and non-survivors among the ICU patients admitted to Al-Kindi MOH Hospital, Baghdad, Iraq.

	Survivors *n* = 351	Non-survivors *n* = 573	*p*-value^a^
**Demographics**			
Age (years), (median, IQR)	55 (46, 65)	61 (53, 70)	<0.001
*Missing data*			
Sex, *n* (%)			
Male	187 (53%)	358 (62%)	0.006
**Pre-existing medical conditions** ^**b**^ **, *n* (%)**			
At least one pre-existing condition	166 (47%)	345 (60%)	<0.001
Cardiovascular disease	122 (35%)	232 (40%)	0.082
Diabetes	84 (24%)	176 (31%)	0.026
Hypertension	85 (24%)	161 (28%)	0.2
Obesity	38 (11%)	62 (11%)	>0.9
Renal disease	7 (2.0%)	26 (4.5%)	0.043
Lung disease	9 (2.6%)	23 (4.0%)	0.2
Cerebrovascular disease	4 (1.1%)	23 (4.0%)	0.012
Other	11 (3.1%)	27 (4.7%)	0.2
**No. of pre-existing medical conditions, *n* (%)**			< 0.001
None	185 (53%)	228 (40%)	
One	57 (16%)	122 (21%)	
Two or more	109 (31%)	223 (39%)	
**COVID-19 diagnosis, *n*(%)**			
PCR result			0.016
Negative PCR Result	75 (26%)	86 (18%)	
Positive PCR Result	218 (74%)	385 (82%)	
*Missing data*	58	102	
CT Scan result			0.081
Non-suggestive of COVID-19	125 (38%)	242 (44%)	
Suggestive of COVID-19	202 (62%)	305 (56%)	
*Missing data*	24	26	
**ICU procedures, *n* (%)**			
At least one ICU procedure	304 (87%)	528 (92%)	0.006
Prone positioning	300 (85%)	524 (91%)	0.005
Thoracostomy tube	6 (1.7%)	12 (2.1%)	0.7
Blood transfusion	4 (1.1%)	10 (1.7%)	0.5
Central line	5 (1.4%)	9 (1.6%)	0.9
Enternal nutrition	3 (0.9%)	9 (1.6%)	0.6
Parenteral nutrition	0 (0%)	5 (0.9%)	0.2
Other procedures^c^	4 (1.1%)	13 (2.3%)	0.2
**ICU complications, *n* (%)**			
At least one complication	44 (13%)	126 (22%)	<0.001
Nasal sore	31 (8.8%)	103 (18%)	<0.001
Bed sore	15 (4.3%)	40 (7.0%)	0.091
Urinal catheter infection	6 (1.7%)	16 (2.8%)	0.3
Ventilator-associated pneumonia (VAP) (clinical)	2 (0.6%)	14 (2.4%)	0.034
Equipment failure	1 (0.3%)	3 (0.5%)	>0.9
Central line infection	1 (0.3%)	1 (0.2%)	>0.9

a Kruskal-Wallis rank sum test; Pearson’s Chi-squared test; Fisher’s exact test.

b Each patient could have more than one pre-existing condition, so the sum of percentages may not add up to 100.

c Other procedures include hemodialysis (*n* = 4) and NG tube insertion (*n* = 1), the remaining other procedures were not captured comprehensively in the study database.

**Figure 4:**
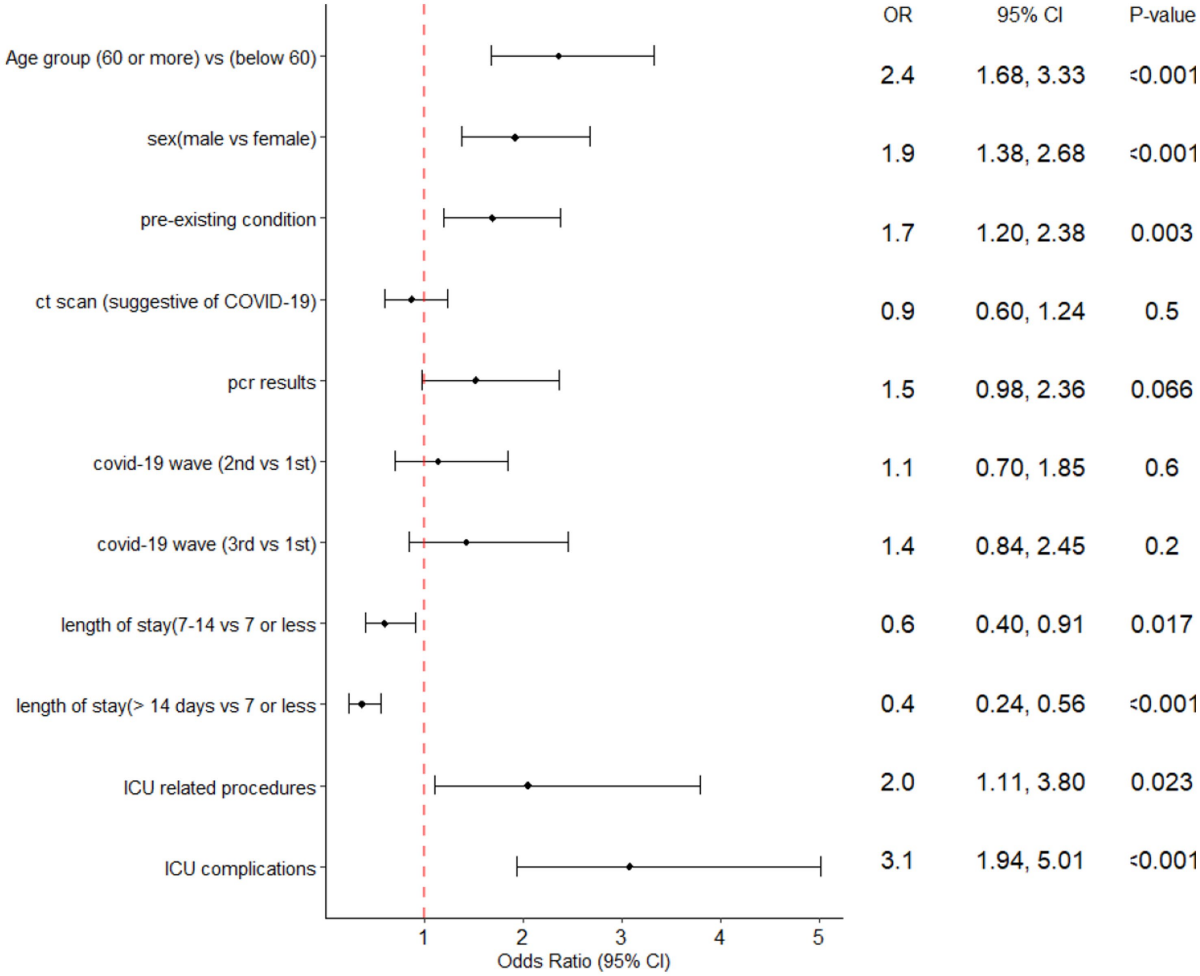
Forest plot of the multiple logistic regression analysis with ICU mortality predictors of ICU patients admitted at Al-Kindi Hospital, Baghdad Iraq.

A multiple logistic regression analysis showed that being 60 years or older, male, and having a pre-existing condition, undergoing aware prone positioning or other ICU procedures, and experiencing ICU complications were all significantly associated with increased odds of ICU mortality (Figure 4).

**Figure 5:**
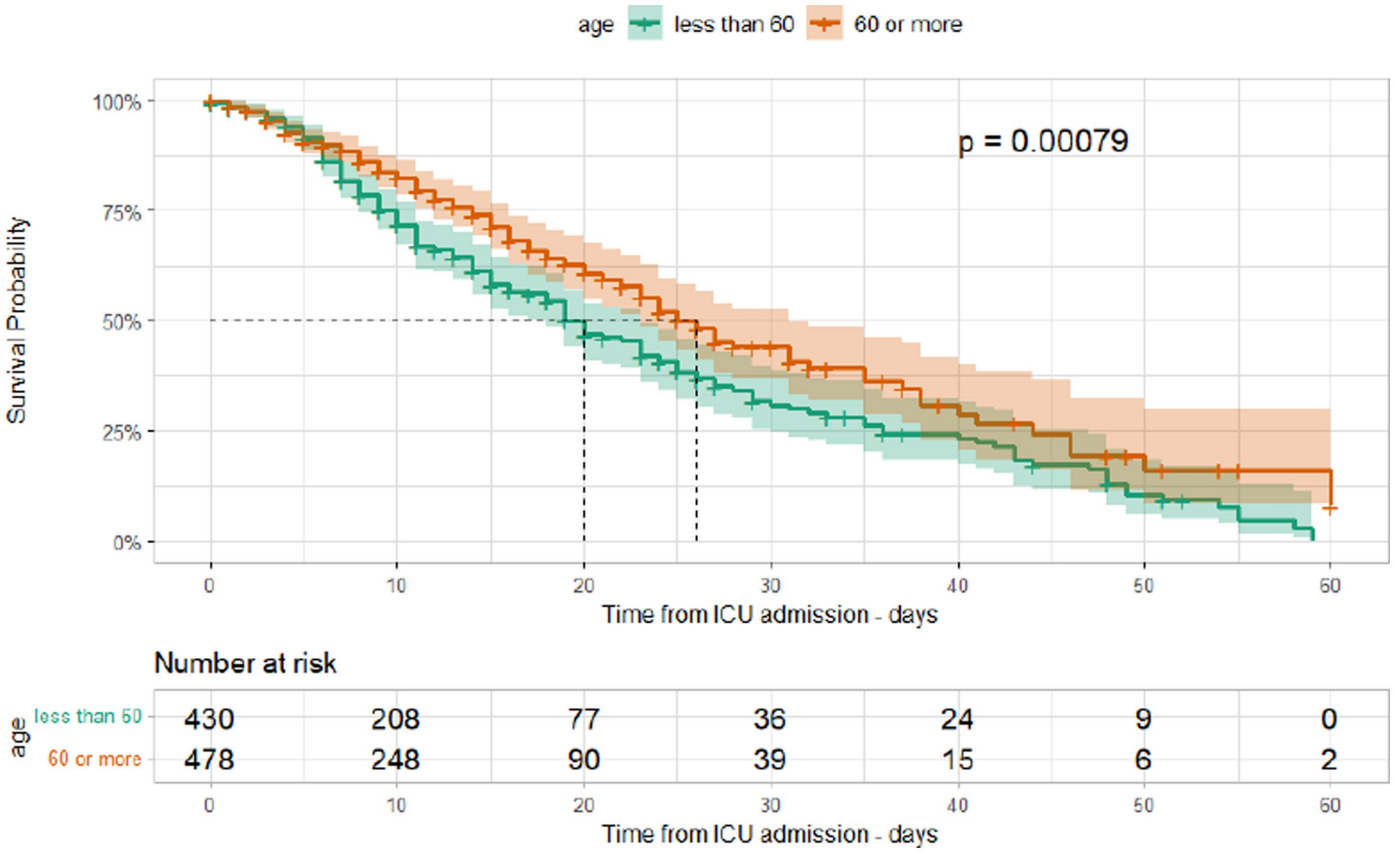
Kaplan–Meier survival curves according to age group in ICU patients admitted at Al Kindi Hospital Baghdad Iraq.

**Figure 6:**
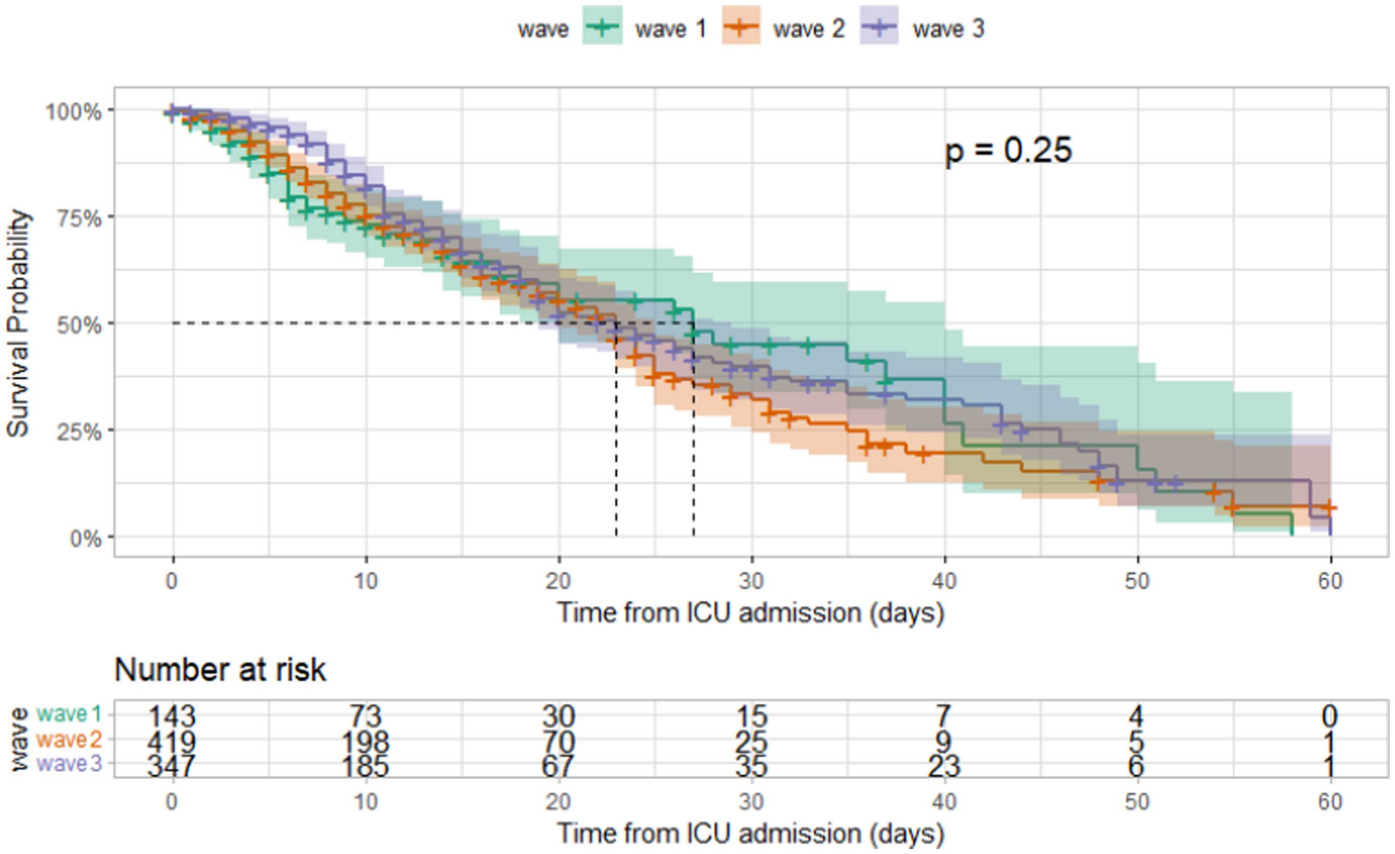
Kaplan–Meier survival curves according to covid-19 wave in ICU patients admitted at Al Kindi Hospital Baghdad Iraq.

The median duration between ICU admission and discharge was 11 days (IQR [6, 20]) and that between ICU admission and mortality was 9 days (IQR [5, 15]). Kaplan–Meier curves for different age groups and among those admitted during different waves are shown in Figures 5, 6. The median hospital stay was longer for patients aged 60 or older (10 days, IQR[5–17]) compared to younger patients (9 days, IQR[5–16] log-rank test *p* < 0.001), but there was no statistically significant difference in the length of stay between patients admitted during different waves (log-rank test *p* = 0.25).

## Discussion

In this study, the clinical features and outcomes of COVID-19 intensive care unit (ICU) patients who received non-invasive mechanical ventilation at a Ministry of Health (MOH) hospital in Baghdad, Iraq were analyzed. Results showed a considerable ICU mortality rate reaching 63%. We demonstrated that male sex, older age, pre-existing medical conditions, shorter ICU stay, ICU procedures and complications during admission were independent predictors of ICU mortality. After adjusting for baseline and clinical characteristics, no significant change in mortality rates was observed among our study participants between different waves of the COVID-19 epidemic in Iraq.

Globally, poor outcomes and elevated mortality rates of COVID-19 patients in the ICU have been widely reported, with a range of 10–78% (1, 23). An updated meta-analysis of observational studies indicated higher mortality rates during the early months of the pandemic followed by improved outcomes attributed to better therapeutics and clinical management (1). The initial reports from China showed a case-fatality rate of 49% among ICU patients. Similarly, high ICU mortality rates were also reported in high-income settings, such Europe and the United States (24–26).

Despite the limited studies on COVID-19 ICU patients in the Middle East, the high ICU mortality rate observed in our study can be compared to other reported rates in the region of similar contextual challenges. For example, a report from MSF in conflict settings of Yemen revealed an ICU mortality rate of 68%, while a multi-center study in Libya reported an ICU mortality rate of 60.4% (27, 28). In Jordan, a hospital treating ICU COVID-19 patients had a much higher mortality rate of 93.8% among their ICU patients (29). In contrast, an ICU in Egypt had a much lower mortality rate of 24.4% (30) and Lebanon reported 55% among their ICU patients (31). Our results also demonstrate a higher mortality rate compared to other low-resource settings in Africa, which reported an ICU mortality rate of 48.2% (32). Possible reasons behind the variations in ICU mortality rates may include differences in clinical management, access to therapeutics, healthcare infrastructure and staff training in Iraq versus other low-resource settings. Additionally, variations in the prevalence of comorbidities, socioeconomic factors and cultural practices might also contribute to these disparities.

In line with previous studies, several factors were found to be predictors of ICU mortality, including age, male sex, and pre-existing medical conditions (6, 7, 9, 33). Moreover, the presence of complications during hospital admission was also a significant predictor of ICU mortality. The most common complications observed were nose and bed pressure ulcers, which can arise due to prolonged ICU stay, the use of non-invasive ventilation *via* a mask, and prone positioning (34). Our study found that awake prone positioning, which was performed for a majority of patients, was associated with increased risk of mortality. Nevertheless, other studies have demonstrated that awake prone positioning can be beneficial for patients with Acute Respiratory Distress Syndrome (ARDS) and can reduce the need for intubation among severe cases (35). Due to resource constraints, the ICU in Baghdad did not provide invasive ventilation, hence, we could not measure the impact of awake prone positioning and non-invasive ventilation in reducing the need for intubation in our study cohort. Despite these limitations, the provision of non-invasive ventilation has been widely recognized as a crucial treatment in improving outcomes and reducing the need for intubation (36, 37).

In this study, a high mortality rate was consistently observed among ICU patients with COVID-19 infection despite improvements in critical care practices such as providing ICU training, increasing bed capacity, improving triage, and implementing stringent admission criteria. While such improvements could have played a role in reducing the number of complications and preventing a further increase in the mortality rate despite an increase in bed capacity, we did not observe a significant decrease in mortality during the second and third waves as we had anticipated. The adjusted odds ratio (OR) did not reveal a significant change in the risk of mortality across the three waves of the pandemic. The persistent high mortality rate may be attributed to factors such as resource constraints and a shortage of ICU beds, leading to delayed admission and prolonged wait times for critical care (38–40). Similar scenarios have been observed in other settings and has been shown to be associated with increased mortality due to increased hospital load and strains on critical care capacity (28, 32, 41).

This study had several limitations, including a lack of comprehensive data from medical records and a lack of information on treatment, laboratory findings, and other clinical indicators that could have provided further insight into the results. Additionally, the analysis did not include non-invasive ventilation due to a lack of data on its provision and timing. The impact of high ICU occupancy rates and admission delays was not evaluated as these details were not recorded. Although the MSF team attempted to improve the performance of the ICU, it was not possible to establish a clear connection between changes in clinical management and patient outcomes. Moreover, severity of illness was not included in the analysis since it was not assessed using validated scoring systems and may have resulted in a biased classification. Additionally, vaccination availability and coverage during the study period were limited, with the majority of the population remaining unvaccinated, hence, vaccination status of patients in this study was not consistently recorded and could not be included in this analysis.

## Conclusion

In low and middle income settings, optimizing ICU care can be challenging due to limited resources and infrastructure. The results of this retrospective observational study demonstrate that despite these challenges, advancements in capacity and clinical practices can significantly mitigate complications in critically ill COVID-19 patients. However, the study also highlights the persistent challenge of high mortality rates in these contexts, with a rate of at least 59% observed throughout the three waves of the pandemic between 2020 and 2021. This necessitates the urgent need for interventions to address this issue and improve patient outcomes.

To address these challenges, it is crucial to prioritize the implementation of effective data collection systems, which can help monitor clinical presentations and identify gaps in clinical management. Although resource limitations may pose barriers to effective data collection, addressing this challenge is essential for improving patient outcomes and identifying opportunities for improvement in ICU care. As the COVID-19 pandemic continues to affect low and middle income settings, it is imperative that we continue to invest in optimizing ICU care and addressing the unique challenges of these contexts.

## Data availability statement

The raw data supporting the conclusions of this article will be made available by the authors, without undue reservation.

## Ethics statement

The studies involving human participants were reviewed and approved by the Research Committee of the National Centre for Training and Human at the Ministry of Health and Environment, Baghdad, Iraq on 31 May 2022 (protocol number 10/2022) and was exempt from the MSF Ethics Review Board following the approval of the Medical Director. Written informed consent from the participants' legal guardian/next of kin was not required to participate in this study in accordance with the national legislation and the institutional requirements.

## Author contributions

RM, AH, UD, KP, and AG contributed to the study conception and design. RM, AH, KA, EZ, and AG developed the study protocol. RM and AH managed the data and did the statistical data analysis. UD and IS performed field data collection. All authors contributed to the article and approved the submitted version.

## Funding

Médecins Sans Frontières funded this study and provides core funding to Epicentre. Médecins Sans Frontières, the funder of this study, participated in the design of the study, the collection and analysis of the data and the writing of this manuscript.

## Conflict of interest

The authors declare that the research was conducted in the absence of any commercial or financial relationships that could be construed as a potential conflict of interest.

## Publisher’s note

All claims expressed in this article are solely those of the authors and do not necessarily represent those of their affiliated organizations, or those of the publisher, the editors and the reviewers. Any product that may be evaluated in this article, or claim that may be made by its manufacturer, is not guaranteed or endorsed by the publisher.

## Figures and Tables

**Table:** 

ARDS	Acute Respiratory Distress Syndrome
CI	Confidence Interval
COVID-19	Coronavirus disease 2019
CPAP	Continuous positive airway pressure
CT	Computerized Tomogram
ER	Emergency Room
HICs	High-income countries
ICU	Intensive Care Unit
IMV	Invasive mechanical ventilation
IPC	Infection Prevention and Control
IQR	Interquartile range
LMICs	Low- and middle- income countries
LOS	Length of stay
MOH	Ministry of Health
MSF	Médecins Sans Frontières
OR	Odds Ratio
RT-PCR	Reverse transcription-polymerase chain reaction
STROBE	Strengthening the reporting of observational studies in epidemiology
VAP	Ventilator-associated pneumonia
WHO	World Health Organization
